# Propionate-producing bacteria in the intestine may associate with skewed responses of IL10-producing regulatory T cells in patients with relapsing polychondritis

**DOI:** 10.1371/journal.pone.0203657

**Published:** 2018-09-20

**Authors:** Jun Shimizu, Takao Kubota, Erika Takada, Kenji Takai, Naruyoshi Fujiwara, Nagisa Arimitsu, Masanori A. Murayama, Yuji Ueda, Sueshige Wakisaka, Tomoko Suzuki, Noboru Suzuki

**Affiliations:** 1 Department of Immunology and Medicine, St. Marianna University School of Medicine, Kawasaki, Japan; 2 Department of Medicine, the Japan Self Defense Forces Central Hospital, Tokyo, Japan; University of Illinois at Urbana-Champaign, UNITED STATES

## Abstract

Relapsing polychondritis (RP) is an inflammatory disease of unknown causes, characterized by recurrent inflammation in cartilaginous tissues of the whole body. Recently, researchers have reported that, in mouse experiments, altered gut microbe-dependent T cell differentiation occurred in gut associated lymphoid tissues. Here, we investigated whether gut microbe alteration existed, and if so, the alteration affected peripheral T cell differentiation in patients with RP. In an analysis of gut microbiota, we found increased annotated species numbers in RP patients compared with normal individuals. In the RP gut microbiota, we observed several predominant species, namely *Veillonella parvula*, *Bacteroides eggerthii*, *Bacteroides fragilis*, *Ruminococcus bromii*, and *Eubacterium dolichum*, all species of which were reported to associate with propionate production in human intestine. Propionate is a short-chain fatty acid and is suggested to associate with interleukin (IL)10-producing regulatory T (Treg) cell differentiation in gut associated lymphoid tissues. IL10 gene expressions were moderately higher in freshly isolated peripheral blood mononuclear cells (PBMC) of RP patients than those of normal individuals. Six hours after the initiation of the cell culture, regardless of the presence and absence of mitogen stimulation, IL10 gene expressions were significantly lower in RP patients than those in normal individuals. It is well known that PBMC of patients with autoimmune and inflammatory diseases show hyporesponsiveness to mitogen stimulation. We suggest that, in RP patients, continuous stimulation of intestinal T cells by excessive propionate leads to the spontaneous IL10 production and a subsequent refractory period of T cells in patients with RP. The hyporesponsiveness of Treg cells upon activation may associate with inflammatory cytokine production of PBMC and subsequently relate to chondritis in RP patients.

## Introduction

Relapsing polychondritis (RP) is an uncommon systemic connective tissue disorder characterized by recurrent and episodic inflammation of cartilaginous tissues, such as ear, nose, joint, and respiratory tract [1]. RP often affects other proteoglycan rich organs, namely eye, inner ear, heart, blood vessels, and kidney [1]. In our survey [2], half of RP patients suffered from laryngotracheal involvement which was generally considered as a major cause of RP morbidity and mortality through infection, tracheomalacia, and so on.

There are no specific laboratory tests for the diagnosis and assessment of disease activity in RP. In our study of Japanese RP patients [2], elevated erythrocyte sedimentation rates and increased serum C-reactive protein concentrations were observed in 68.2% and 86.2% of the patients, respectively.

The initial investigation of autoantibodies against type II collagen reported that the recognition manners to the antigens were disease (RP)-specific and the serum levels were correlated with disease severity [3]. Autoantibodies to matrilin, another cartilage protein, were observed in RP patients and had affinity to tracheolaryngeal and nasal cartilage [4].

T helper type 1 (Th1)-skewed responses were found in the serum cytokine concentrations of RP patients [5]. As an inflammatory disease with enhanced Th1 responses, anti-tumor necrosis factor (TNF)α antibody administration was suggested to be a choice of treatment in patients with refractory RP [6]. Th1-skewed responses were observed in natural killer T cells, a subset of innate-like lymphocytes, in RP patients [7].

Recent studies demonstrated that Th17 cells and regulatory T (Treg) cells play a crucial role in human diseases [8]. Th17 cells and Treg cells exhibited proinflammatory and antiinflammatory effects, respectively, on the other types of immunocompetent cells [8]. Both of the Th cell subpopulations were reported to differentiate in gut associated lymphoid tissues and need several specified gut microbes for their differentiation [9,10]. Researchers suggested that gut microbes were able to modulate the balance between Th17 cells and Treg cells through the metabolites, short-chain fatty acids [11–18]. Depletion of short-chain fatty acids, such as propionate and butyrate, was thought to cause various human diseases, such as type 1 diabetes, asthma, and multiple sclerosis through the unbalance of Th17 and Treg cells [16–18]. Actually, we previously demonstrated that compositional alteration of gut microbes and skewed responses of T cells in the peripheral blood occurred in patients with RP [19].

Here, we obtained 16S rRNA metagenomic data of RP patients and compared the data with those of normal individuals. We analyzed gene expressions of inflammation associated cytokines in freshly isolated and cultured peripheral blood mononuclear cells (PBMC) in RP patients and normal individuals for comparison.

## Materials and methods

### Ethics statement

This study was approved by the institutional review boards of St. Marianna University School of Medicine and was registered with the University Hospital Medical Information Network-Clinical Trials Registry (UMIN000018937). We conducted our research according to the principles expressed in the Declaration of Helsinki. We obtained written informed consent from each individual prior to enrolment in the study.

### 16S rRNA metagenomic data

We analyzed the feces of 25 RP patients and 27 normal individuals to obtain 16S rRNA metagenomic data. Mean age (years) of the patients was 54.8 ± 2.8 (standard error of the means) and that of normal individuals was 52.8 ± 2.8. The male to female ratios were 7:18 in RP patients and 12:15 in normal individuals. In patients, mean age of disease onset was 44.8 ± 3.4 and mean disease duration (years) was 10.0 ± 1.6. The clinical data of the 25 RP patients were summarized in Table 1.

**Table 1:** Clinical characteristics of patients with relapsing polychondritis.

**% Organ involvement**				
	**Metagenomic analysis**^a^ **(n = 25)**		**Gene expression analysis**^a^ **(n = 22)**	
	**At onset**	**Cumulative**	**At onset**	**Cumulative**
Ear	60.0	60.0	68.2	68.2
Nose	44.0	44.0	59.1	59.1
Airway	48.0	48.0	40.9	40.9
Inner ear	8.0	20.0	4.6	13.6
Joint	24.0	32.0	18.2	36.4
Eye	36.0	52.0	40.9	54.6
Skin	0	4.0	0	4.6
Cardiovascular	0	8.0	0	13.6
Central nervous system	0	4.0	0	0
Renal	0	8.0	0	9.1
**Medications at the time of sample collection, %**				
	**Metagenomic analysis**^a^ **(n = 25)**		**Gene expression analysis**^a^ **(n = 22)**	
Steroid	88.0	86.3
Methotrexate	28.0	36.4
Cyclophosphamide	0	0
Cyclosporine	24.0	18.2
Tacrolimus	16.0	18.2
Azathioprine	4.0	4.6
Mizoribine	8.0	4.6
Biologic agents	20.0	31.8

^a^18 patients were evaluated with both the metagenomic analysis and the gene expression analysis.

We described here the study methods briefly. We extracted genomic DNA from fecal samples by treating them with achromopeptidase (Wako Pure Chemical Industries, Tokyo, Japan) [20]. We amplified the V1–V2 16S rRNA gene region by primers according to a procedure reported previously [21]. We purified (AMPure XP magnetic purification beads, Beckman Coulter, Tokyo, Japan), quantified (Agilent 2100 Bioanalyzer, Agilent Technologies Japan, Tokyo, Japan), and sequenced the amplicon libraries (Ion Torrent PGM, Life Technologies Japan, Tokyo, Japan).

### Sequence analysis

We filtered the output file using QIIME software (version 1.9.1) with the default settings. After calculation of an operational taxonomic unit (OTU) table, we estimated microbial α and β diversity using QIIME software.

At the same time, we obtained another OTU table by QIIME software with a Greengenes-formatted database. We modified and predicted the file by using PICRUSt software and obtained a taxonomy file and a bacterial gene function file. We have uploaded the files to the Galaxy (http://huttenhower.sph.harvard.edu/galaxy) [22].

### Statistical analysis of the sequenced data

Uploaded files were analyzed statistically with LEfSe (linear discriminant analysis (LDA) effect size) software [23]. LEfSe was an algorithm to find significant differences in genomic features (genes, pathways, or taxa) among groups, using nonparametric factorial Kruskal-Wallis sum-rank test and Wilcoxon rank-sum test. In LEfSe software, linear discriminant analysis was used to estimate the effect size of each differentially abundant feature. Those features that showed higher log LDA scores than 2.0 were chosen for subsequent plotting of output charts. Consequently, LEfSe indicated those features that better discriminated among groups with the log LDA scores.

### Cell culture, gene expression analyses, and an Elisa assay

We obtained PBMC of 22 RP patients and 11 normal individuals. 18 patients were evaluated with both the metagenomic analysis and the gene expression analysis. Mean age (years) of the patients was 53.3 ± 3.4 (standard error of the means) and that of normal individuals was 43.3 ± 2.8. The male to female ratios were 8:14 in RP patients and 5:6 in normal individuals. In patients, mean age of disease onset was 42.5 ± 4.2 and mean disease duration (years) was 10.5 ± 1.6. The clinical data of the 22 RP patients were summarized in Table 1.

PBMC were cultured for 24 hours with and without 1 μg/ml phytohemagglutinin (PHA, Sigma-Aldrich, St. Louis, MO) and a low concentration of phorbol myristate acetate (PMA, Sigma-Aldrich, 4 ng/ml). We harvested the cells and extracted total RNA from freshly isolated, 6-hour-cultured, and 24-hour-cultured PBMC. We studied 5 combinations of TaqMan primers and probes from Life Technologies Japan Ltd. (Tokyo, Japan) as follows: interleukin (IL)10, forkhead box P3 (Foxp3), IL1β, IL6, and TNFα. Gene expressions of RP patients were estimated by the ΔΔCt method, followed by normalization of the titers relative to 2^-ΔΔCt^ of normal individuals. The gene expression levels of normal individuals were defined as 1.000.

We measured fecal secretory IgA (sIgA) of 25 RP patients and 24 normal individuals using an Elisa kit (Eagle Biosciences, Nashua, NH).

### Statistical analysis of demographical, gene expression, Elisa assay, and metagenomic data

We compared demographical data of patients with those of normal individuals by using Wilcoxon rank-sum test or Fisher’s exact test. We compared OTU numbers, α diversity index scores, relative gene expressions, and Elisa titers by using Wilcoxon rank-sum test with statistical software JMP 13.0.0 (SAS Institute Japan, Tokyo, Japan).

## Results

### Increased relative abundance of propionate-producing bacteria in patients with RP

We analyzed the feces of 25 RP patients and 27 normal individuals to obtain 16S rRNA metagenomic data. We compared the data of RP patients with those of age- and gender-matched normal individuals.

**Fig 1:**
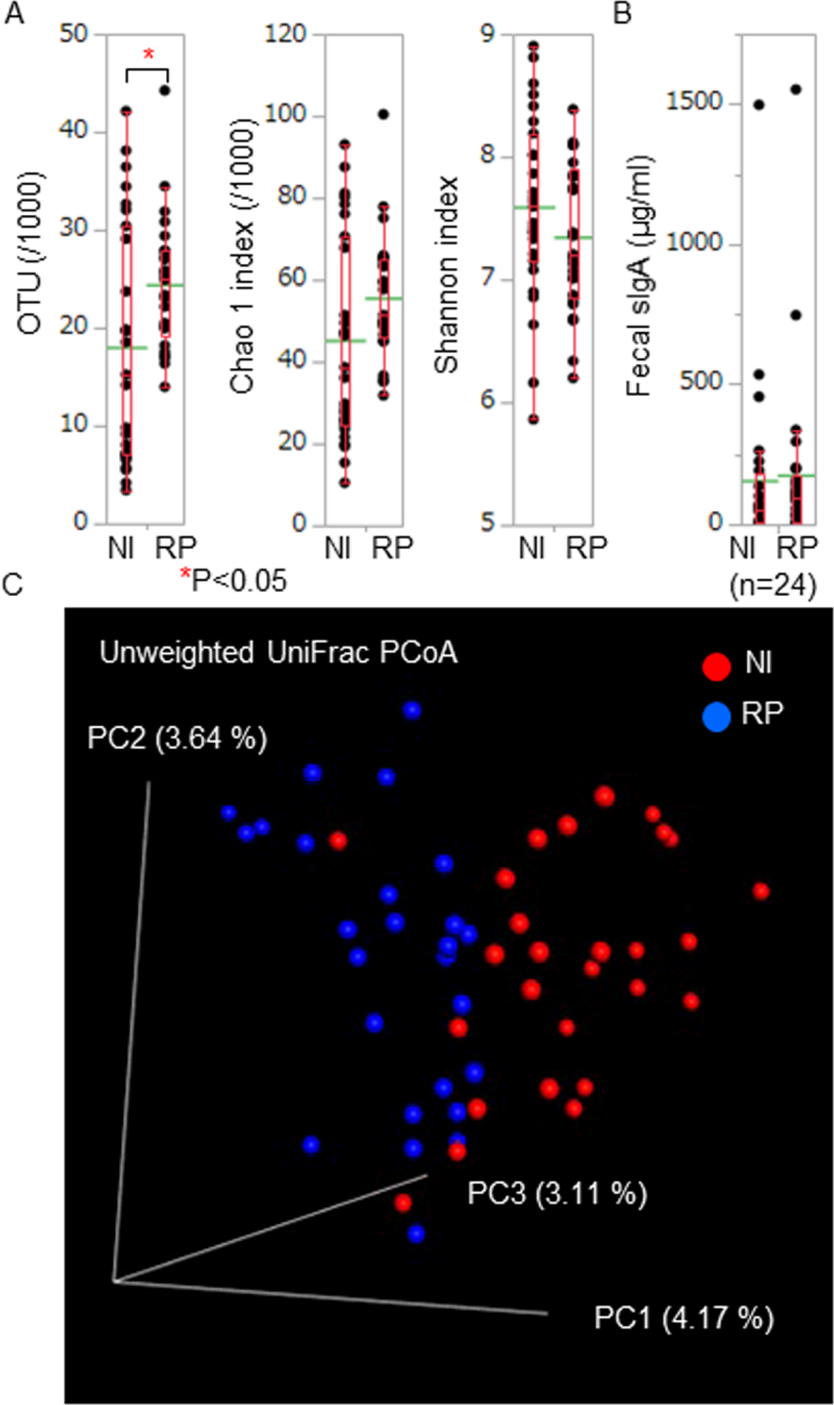
Bacterial numbers and diversity of RP gut microbiota. (A) We estimated OTU numbers (annotated species numbers) and α diversity scores (Chao 1 and Shannon indexes) of each sample. OTU numbers of RP patients (RP) were significantly higher than those of normal individuals (NI). Chao 1 and Shannon index scores were comparable between RP patients and normal individuals. (B) We measured fecal sIgA concentrations and compared the data between RP patients and normal individuals. Fecal sIgA concentrations were comparable between RP patients and normal individuals. These biological parameters of RP patients and normal individuals were displayed with dot plots. A box-plot and a mean level (green line) of each group of RP patients and normal individuals were indicated. (C) We estimated β diversity between RP patients and normal individuals. We showed the PCoA plots in a three dimensional structure where three axes and each contribution ratio were depicted. We exploratory evaluated the distance between the distribution of RP patients and that of normal individuals using two-sided Student's two-sample t-test and Monte Carlo permutations. We obtained a significant P value of the β diversity between RP patients and normal individuals in unweighted UniFrac PCoA (P = 0.01).

We estimated OTU numbers (annotated species numbers) and α diversity (Chao 1 and Shannon indexes) using QIIME software. α diversity was defined as the diversity within a community and Chao 1 and Shannon indexes were species richness and evenness estimators, respectively. OTU numbers were significantly high in RP patients compared with those in normal individuals (Fig 1A). Chao 1 and Shannon index scores were comparable between RP patients and normal individuals (Fig 1A). Fecal sIgA concentrations, which may suggest activation of intestinal immunity, were comparable between RP patients and normal individuals (Fig 1B).

We exploratory estimated β diversity between RP patients and normal individuals. β diversity was defined as the diversity between communities. We obtained a figure of principal coordinates analysis (PCoA) plots using a linear conversion formula (Fig 1C).

We evaluated the distance between the distribution of RP patients and that of normal individuals using two-sided Student's two-sample t-test and Monte Carlo permutations of QIIME software. We obtained a significant P value of the β diversity between RP patients and normal individuals in unweighted UniFrac PCoA (P = 0.01), suggesting that the gut microbe compositional difference was obvious between RP patients and normal individuals.

We analyzed the metagenomic data of bacterial taxa using LEfSe analytic method to detect major taxon differences between RP patients and normal individuals. There were significant differences in relative abundance of 25 bacterial species between RP patients and normal individuals (Table 2, S1 and S2 Figs).

**Table 2:** Abundant bacterial species in RP patients and normal individuals.

Abundant species in RP patients	Abundant species in normal individuals
*Eubacterium dolichum*★	*Serratia* species
*Staphylococcus* species	*Leuconostoc* species
*Coprobacillus cateniformis*	*Salmonella enterica*
*Bifidobacterium bifidum*	*Roseburia faecis*
*Ruminococcus bromii*★	*Pseudomonas* species
*Acidaminococcus* species★	
*Blautia product*	
*Lactobacillus vaginalis*	
*Christensenella* species★	
*Bifidobacterium animalis*	
*Proteus* species	
*Gardnerella* species	
*Anaerofustis* species	
*Bacteroides eggerthii*★	
*Finegoldia* species	
*Kocuria rhizohila*	
*Bacteroides fragilis*★	
*Veillonella parvula*★	
*Scardovia* species	
*Lactobacillus salivarius*	

★, These species were reported to associate with propionate production in the intestine.

Relative abundance of (in the phylum *Firmicutes*) *Eubacterium dolichum*, *Staphylococcus* species, *Coprobacillus cateniformis*, *Ruminococcus bromii*, *Acidaminococcus* species, *Blautia producta*, *Lactobacillus vaginalis*, *Christensenella* species, *Anaerofustis* species, *Finegoldia* species, *Veillonella parvula*, *Lactobacillus salivarius*, (in the phylum *Actinobacteria*) *Bifidobacterium bifidum*, *Bifidobacterium animalis*, *Gardnerella* species, *Kocuria rhizohila*, (in the phylum *Bacteroidetes*) *Bacteroides fragilis*, *Scardovia* species, *Bacteroides eggerthii*, and (in the phylum *Proteobacteria*) *Proteus* species increased significantly in patients with RP.

In the RP predominant bacteria, several species (indicated in Table 2 using asterisks) were reported to associate with propionate production in the intestine (details were described in the Discussion section) [24–31].

Relative abundance of (in the phylum *Firmicutes*) *Leuconostoc* species, *Roseburia faecis*, (in the phylum *Proteobacteria*) *Serratia* species, *Salmonella enterica*, and *Pseudomonas* species increased significantly in normal individuals.

### Abundant Kyoto Encyclopedia of Genes and Genomes (KEGG) modules for the citrate cycle in patients with RP

We assigned predicted gene functions of the metagenomic data according to the KEGG module database and identified predominant modules in RP patients and normal individuals (Tables 3 and 4 and S3 Fig). A KEGG module was a collection of manually defined functional units and was utilized for the annotation and biological interpretation of sequenced genomes.

**Table 3:** Abundant KEGG modules in RP patients.

KEGG module identifiers	KEGG module names
M00096	C5 isoprenoid biosynthesis, non-mevalonate pathway
M00048	Inosine monophosphate biosynthesis, PRPP + glutamine = > IMP
M00016	Lysine biosynthesis, succinyl-DAP pathway, aspartate = > lysine
M00019	Valine/isoleucine biosynthesis, pyruvate = > valine / 2-oxobutanoate = > isoleucine
M00121	Heme biosynthesis, glutamate = > protoheme/siroheme
M00011	Citrate cycle, second carbon oxidation, 2-oxoglutarate = > oxaloacetate
M00009	Citrate cycle
M00060	Lipopolysaccharide biosynthesis, KDO2-lipid A
M00335	Sec (secretion) system
M00324	Dipeptide transport system
M00012	Glyoxylate cycle
M00008	Entner-Doudoroff pathway, glucose-6P = > glyceraldehyde-3P + pyruvate
M00117	Ubiquinone biosynthesis, prokaryotes, chorismate = > ubiquinone
M00149	Succinate dehydrogenase, prokaryotes
M00229	Arginine transport system
M00150	Fumarate reductase, prokaryotes
M00124	Pyridoxal biosynthesis, erythrose-4P = > pyridoxal-5P
M00260	DNA polymerase III complex, bacteria
M00045	Histidine degradation, histidine = > N-formiminoglutamate = > glutamate
M00198	Putative sn-glycerol-phosphate transport system

**Table 4:** Abundant KEGG modules in normal individuals.

KEGG module identifiers	KEGG module names
M00125	Riboflavin biosynthesis, GTP = > riboflavin/FMN/FAD
M00119	Pantothenate biosynthesis, valine/L-aspartate = > pantothenate
M00185	Sulfate transport system
M00157	F-type ATPase, prokaryotes and chloroplasts
M00164	ATP synthase
M00003	Gluconeogenesis, oxaloacetate = > fructose-6P
M00018	Threonine biosynthesis, aspartate = > homoserine = > threonine
M00115	NAD biosynthesis, aspartate = > NAD
M00026	Histidine biosynthesis, PRPP = > histidine
M00200	Putative sorbitol/mannitol transport system
M00349	Microcin C transport system
M00225	Lysine/arginine/ornithine transport system
M00136	GABA biosynthesis, prokaryotes, putrescine = > GABA
M00144	NADH:quinone oxidoreductase, prokaryotes

In the abundant KEGG modules of RP patients, we observed gene functions for glycolysis (M00008), citrate cycle (M00009, M00011, M00012, M00149, M00150), and amino acid biosynthesis and transport (M00016, M00019, M00229).

**Fig 2:**
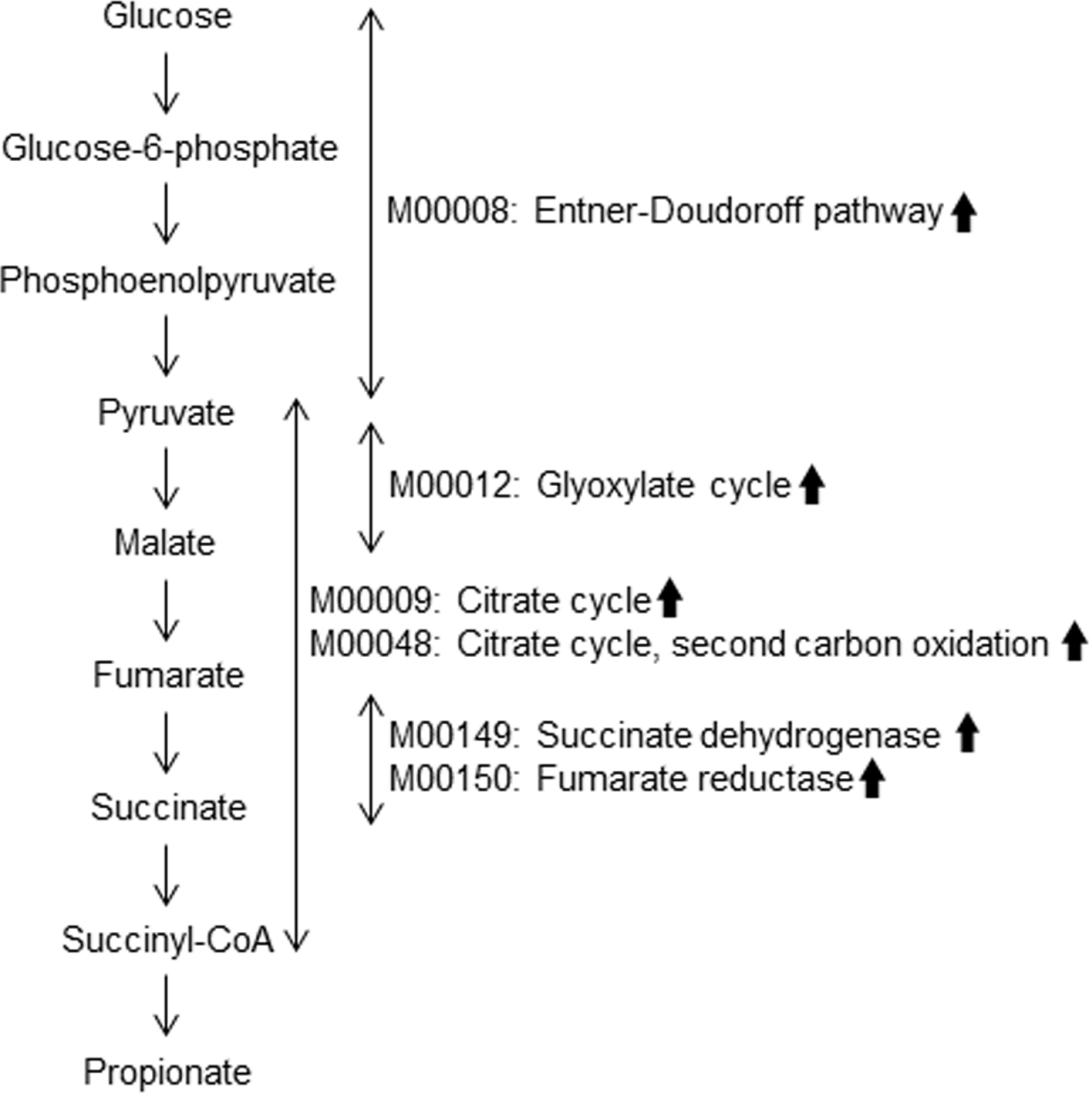
Schematic representation of succinate-propionate pathway and its alterations in patients with RP. Several predominant gene functions of RP gut microbiota of this study (arrows) corresponded to several components of the succinate-propionate pathway. *Veillonella* species, *Bacteroides* species, and *Acidaminococcus* species utilize the pathway for the propionate production.

Fig 2 indicates RP predominant gene functions associated with succinate-propionate pathway [24,26], suggesting greater capacity for propionate production in the intestine of RP patients than that of normal individuals.

In the abundant KEGG modules of normal individuals, we observed gene functions for electron transport system (M00115, M00157, M00164, M00144) and vitamin B biosynthesis (M00125, M00119).

### RP PBMC exhibited marked alterations in IL10 gene expressions

Several RP predominant species were reported to associate with propionate production in the intestine [24–31]. It was suggested that propionate promoted IL10-producing Treg cell differentiation in the intestine with gene expressions of Foxp3 [11,13–15]. We analyzed cytokine gene expressions in PBMC of 22 RP patients and 11 normal individuals to assess whether skewed IL10-producing Treg cell responses existed in patients with RP. PBMC were cultured with and without mitogen stimulation and we extracted messenger RNA from freshly isolated, 6-hour-cultured, and 24-hour-cultured PBMC.

**Fig 3:**
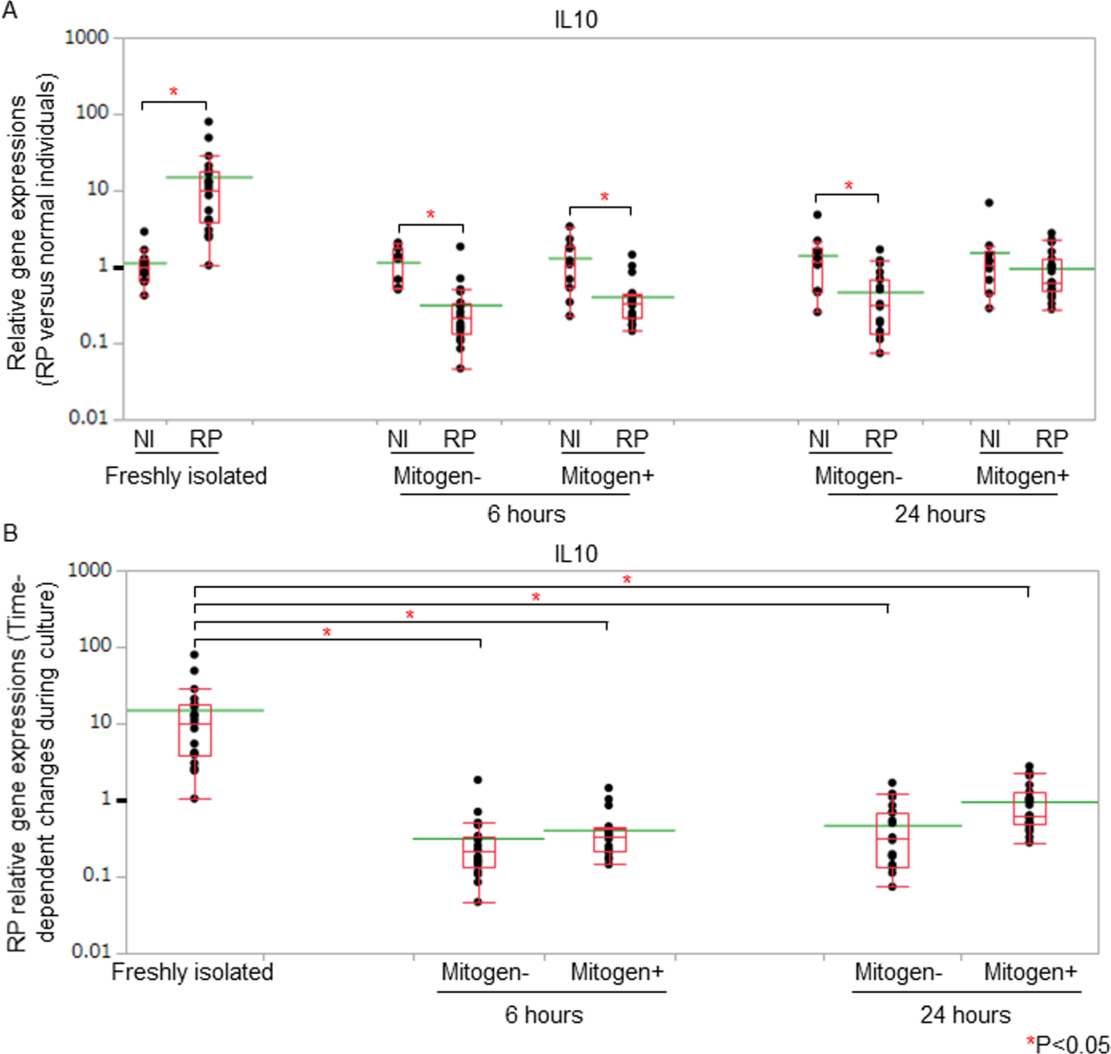
IL10 gene expressions on PBMC of RP patients. (A) IL10 gene expressions of RP PBMC compared with those of normal PBMC. We measured IL10 gene expressions of freshly isolated, 6-hour-cultured, and 24-hour-cultured PBMC in RP patients (RP) and normal individuals (NI). Gene expressions of RP patients were estimated by the ΔΔCt method, followed by normalization of the titers relative to 2^-ΔΔCt^ of normal individuals. The gene expression levels of normal individuals were defined as 1.000. IL10 gene expressions of freshly isolated PBMC were significantly higher in RP patients than those in normal individuals. IL10 gene expressions were significantly lower in RP patients than those of normal individuals 6 hours after the initiation of the cell culture, regardless of the presence and absence of mitogen stimulation. (B) Time-dependent changes of IL10 gene expressions of RP PBMC. We compared the gene expression data of the freshly isolated PBMC with those of cultured PBMC in patients with RP. IL10 gene expressions decreased significantly regardless of the presence and absence of mitogen stimulation in RP patients after the initiation of the cell culture. Relative gene expressions of RP patients against those of normal individuals were displayed with dot plots. A box-plot and a mean level (green line) of each group of RP patients and normal individuals were indicated.

Freshly isolated PBMC elicited excessive responses with regard to IL10 production in RP patients compared with normal individuals. IL10 gene expressions of freshly isolated PBMC were significantly higher in RP patients than those in normal individuals even though the expression levels were moderate (Fig 3A). These data suggested that IL10-producing T cells were activated substantially *in vivo* in patients with RP. IL10 gene expressions were significantly lower in RP patients than those in normal individuals 6 hours after the initiation of the cell culture regardless of the presence and absence of mitogen stimulation (Fig 3A). When we compared the data of the freshly isolated PBMC with those of cultured PBMC in patients with RP, IL10 gene expressions decreased significantly regardless of the presence and absence of mitogen stimulation after the initiation of the cell culture (Fig 3B).

**Fig 4:**
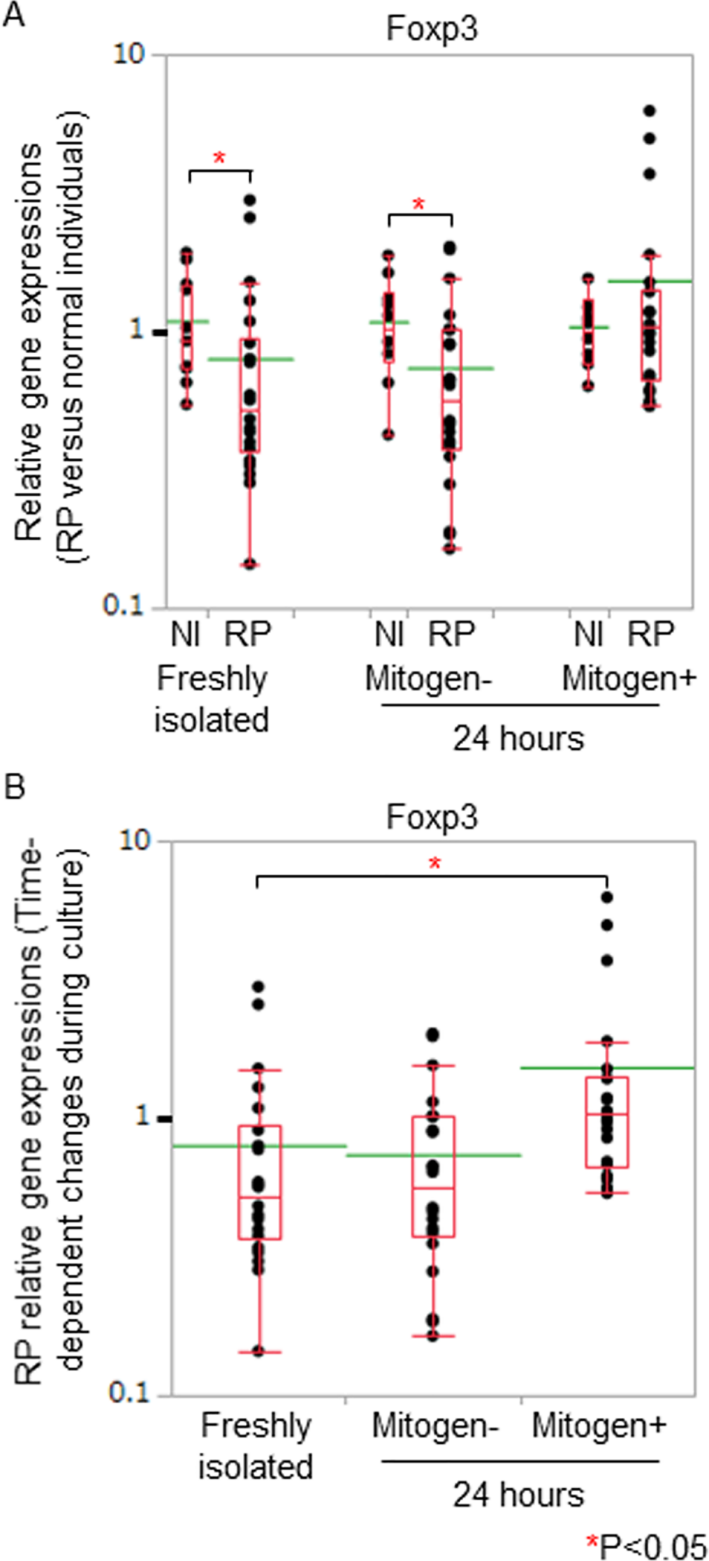
Foxp3 gene expressions in PBMC of RP patients. (A) We assessed RP relative gene expressions of Foxp3 by normalizing the titers to 2^-ΔΔCt^ of normal individuals. The gene expression levels of normal individuals were defined as 1.000. Foxp3 gene expressions of RP freshly isolated PBMC were significantly lower than those of normal individuals. (B) We compared the gene expression data of the freshly isolated PBMC with those of cultured PBMC in patients with RP. Foxp3 gene expressions increased significantly in the presence of mitogen stimulation 24 hours after the initiation of the cell culture. Relative gene expressions of RP patients against those of normal individuals were displayed with dot plots. A box-plot and a mean level (green line) of each group of RP patients and normal individuals were indicated.

Foxp3 gene expressions of RP freshly isolated PBMC were significantly lower than those of normal individuals (Fig 4A). When we compared the gene expression data of the freshly isolated PBMC with those of cultured PBMC in patients with RP, Foxp3 gene expressions increased significantly in the presence of mitogen stimulation 24 hours after the initiation of the cell culture (Fig 4B).

**Fig 5:**
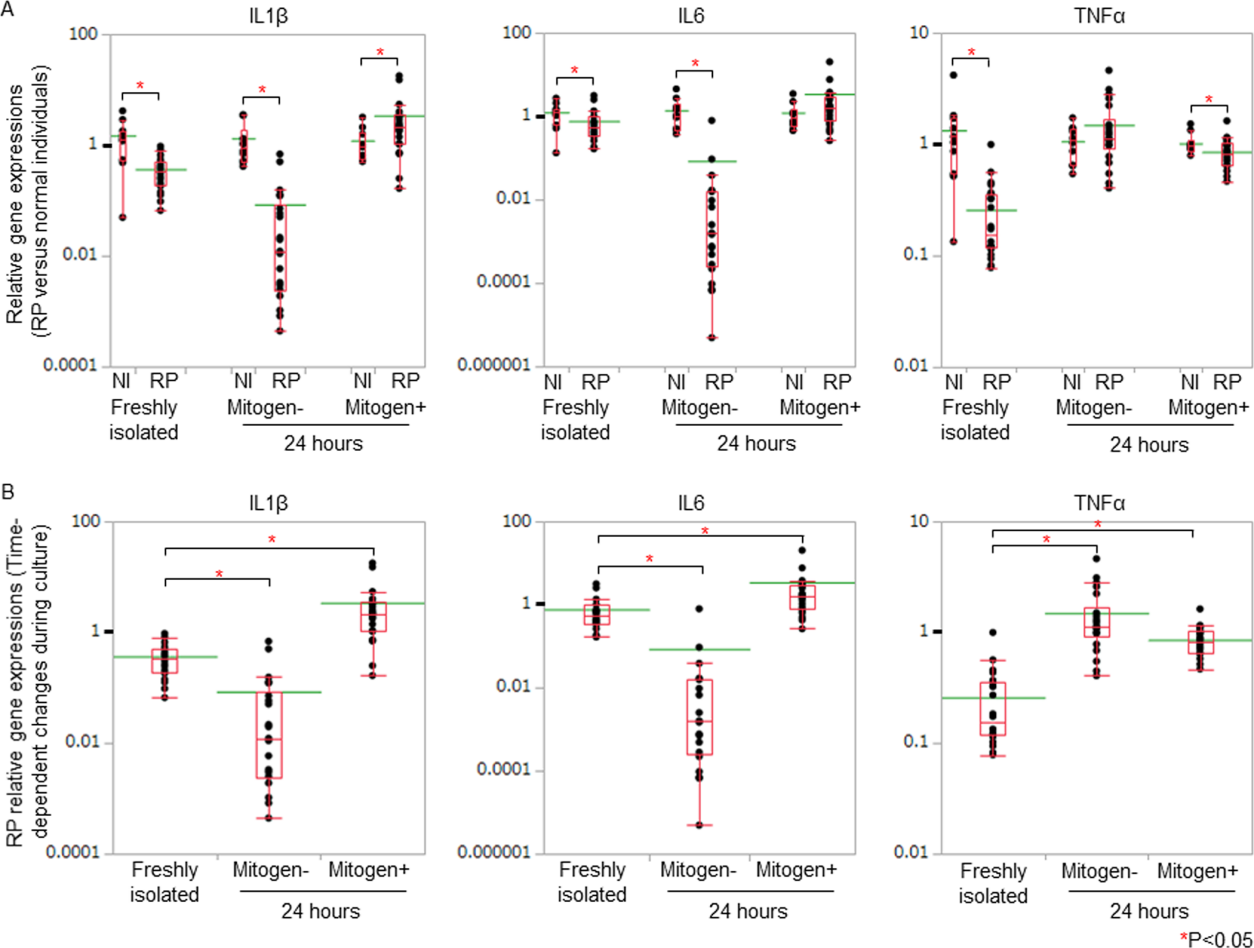
Inflammatory cytokine gene expressions in PBMC of RP patients. (A) We assessed RP relative gene expressions of inflammatory cytokines by normalizing the titers to 2^-ΔΔCt^ of normal individuals. The gene expression levels of normal individuals were defined as 1.000. IL1β gene expressions of RP patients were significantly higher than those of normal individuals in the presence of mitogen stimulation, 24 hours after the initiation of the cell culture. (B) We compared the gene expression data of the freshly isolated PBMC with those of cultured PBMC in patients with RP. After the initiation of the cell culture, in the absence of mitogen stimulation, TNFα gene expressions increased significantly in RP patients. With the stimulation, IL1β, IL6, and TNFα gene expressions increased significantly in RP patients. Relative gene expressions of RP patients against those of normal individuals were displayed with dot plots. A box-plot and a mean level (green line) of each group of RP patients and normal individuals were indicated.

IL1β gene expressions of RP patients were significantly higher than those of normal individuals in the presence of mitogen stimulation, 24 hours after the initiation of the cell culture (Fig 5A). We compared the gene expression data of the freshly isolated PBMC with those of cultured PBMC in patients with RP. After the initiation of the cell culture, in the absence of mitogen stimulation, TNFα gene expressions increased significantly in RP patients (Fig 5B). With the stimulation, IL1β, IL6, and TNFα gene expressions increased significantly in RP patients (Fig 5B).

## Discussion

Here, we observed increased relative abundance of propionate-producing species in the intestine of RP patients. We found skewed Treg cell responses in patients with RP.

*Veillonella parvula*, *Bacteroides eggerthii*, *Bacteroides fragilis* (Table 2) were major propionate-producing bacteria in human intestine [24–28].

The most abundant species of this study in patients with RP, *Eubacteria dolichum* and *Christensenella* species, showed positive correlations with increased propionate concentrations in the human intestine on a resistant starch diet [29]. In other studies, resistant starch feeding significantly elevated fecal propionate concentrations with increased abundance of *Ruminococcus bromii* in the intestine of rats [30] and humans [31]. These data suggested that high concentrations of intestinal propionate produced by RP gut microbes modified the intestinal immune functions.

The gut derived propionate was reported to be metabolized in the host liver and the concentrations in the peripheral blood were low [32,33]. In the intestine, propionate induced IL10-producing Treg cells [11,13–15], but not TGFβ-producing Treg cells [11], through the epigenetic modification [11,12,14,15]. The intestinal propionate did not affect [11] and reduced [13] Th1 and Th17 cell populations of the intestine. Production of inflammatory cytokines, namely IL6 and IL12, by dendritic cells, was reduced by propionate [34] and propionate-treated dendritic cells increased Treg cells in *in vitro* experiments [12,35]. These findings seemed to share several common characteristics with gene expression analysis of this study.

IL10 enhanced gene expression of and protein production of type II collagen in injured cartilage grafts [36]. IL10 increased tibial length in the organ culture of IL10 gene-deficient mouse [37]. Thus inappropriate production of IL10 may bring about degeneration of cartilaginous tissues. Refractory phase in IL10 gene expression of PBMC in the cell culture may promote degeneration of chondrocyte in RP patients with inflammatory conditions. T cell hyporesponsiveness against mitogen stimulation was well known in patients with inflammatory diseases [38,39].

Increased gene expressions of inflammatory cytokines in response to mitogen stimulation became evident (Fig 5B). Mitogen or (auto)antigen may be important for provoking inflammatory cytokine production in patients with RP. In the absence of mitogen stimulation, TNFα gene expressions increased significantly in the 24-hour-culture (Fig 5B). It is known that several lactic acid-producing bacteria, including *Bifidobacterium* species and *Lactobacillus* species [40,41], and the bacterial cell surface components [42] increased TNFα production of human PBMC. Gut microbes of RP patients may associate with increased TNFα production by some means. Further studies are needed to elucidate the underlying mechanisms.

In addition, IL10 hyporesponsiveness of Treg cells in response to mitogen stimulation may associate with TNFα overproduction of PBMC in patients with RP [43]. In regard to the efficiency of anti-TNFα antibody treatment in RP patients [6], TNFα seems to play a crucial role in the development of chondritis.

We observed characteristic alteration of 34 bacterial gene functions in patients with RP and normal individuals (Tables 3, 4, and S3 Fig). In RP patients, we observed abundant gene functions for glycolysis and citrate cycle, several functions of which were important for succinate-propionate pathway (Fig 2) [24]. *Veillonella* species, *Bacteroides* species, and *Acidaminococcus* species utilize the pathway for the propionate production [24–26] and the data support our hypothesis of a possible relationship between RP gut microbiota and T cell dysfunctions in patients with RP.

In normal individuals, we observed abundant gene functions for several proteins of electron transport chain, including ATP. Bacterial ATP was shown to increase Th17 cells in the intestine through the activation of antigen presenting cells which expressed TNFα gene [44]. It is possible that reduced ATP synthesis of RP gut microbes brings about Th17/Treg cell imbalance in the intestine.

Calcineurin inhibitors, namely cyclosporine and tacrolimus, negatively regulate T cell receptor signaling pathway and inhibit Th cell activation [45]. The compounds directly reduced the frequencies and functions of Treg cells, but not Th17 cells [46], in several *in vitro* and *in vivo* assays [46–49]. It may be important to assess peripheral T cell and the host responses to the inhibitors and elucidate the relationships between them in RP patients.

In conclusion, our findings suggested that propionate-producing gut microbes became predominant, leading to defective Treg cell function upon activation in patients with RP. Decreased production of IL10 by Treg cells and increased production of TNFα by PBMC may lead to chondritis in RP patients.

## Supporting information

S1 Fig PICRUSt/LEfSe plots of abundant bacterial taxa in RP patients and normal individuals. We analyzed metagenomic data of bacterial taxa using PICRUSt/LEfSe to assess major taxon differences between RP patients (RP) and normal individuals (NI). PICRUSt/LEfSe provided us with bar plots of prevalent biological features with the log LDA scores (effect sizes).
In this chart, significantly enriched bacterial taxa in samples obtained from RP patients were exhibited by green bars. Significantly enriched bacterial taxa in samples obtained from normal individuals were exhibited by red bars. “p__”, “c__”, “o__”, “f__”, “g__”, and “s__” indicated phylum, class, order, family, genus, and species, respectively.
Predominant species in RP patients and normal individuals were listed in Table 2.
(TIF)

S2 Fig A PICRUSt/LEfSe cladogram of abundant bacterial taxa in RP patients and normal individuals. PICRUSt/LEfSe provided us with a cladogram of seven levels (from kingdom to species) from the same OTU table of S1 Fig. Circles ranged from the phylum (the innermost) to the species.
Significantly enriched bacterial taxa in samples obtained from RP patients (RP) were exhibited by small green circles and green shadings. Significantly enriched bacterial taxa in samples obtained from normal individuals (NI) were exhibited by small red circles and red shadings. The circle sizes corresponded to the log LDA scores (effect sizes).
This cladogram demonstrated that the class Bacilli, the order Actinomycetales, and several genera and species were abundant in RP patients.
(TIF)

S3 Fig Abundant KEGG modules in RP patients and normal individuals. We assigned predicted gene functions according to the KEGG module database to identify predominant modules in RP patients (RP) and normal individuals (NI).
Predominant KEGG modules in RP patients and normal individuals were listed in Tables 3 and 4, respectively.
(TIF)
