# Supplementary figures and images for: Propionate-producing bacteria in the intestine may associate with skewed responses of IL10-producing regulatory T cells in patients with relapsing polychondritis
Source: PLoS One. 2018 Sep 20;13(9):e0203657. doi: 10.1371/journal.pone.0203657 (PMC6147427; doi:10.1371/journal.pone.0203657)

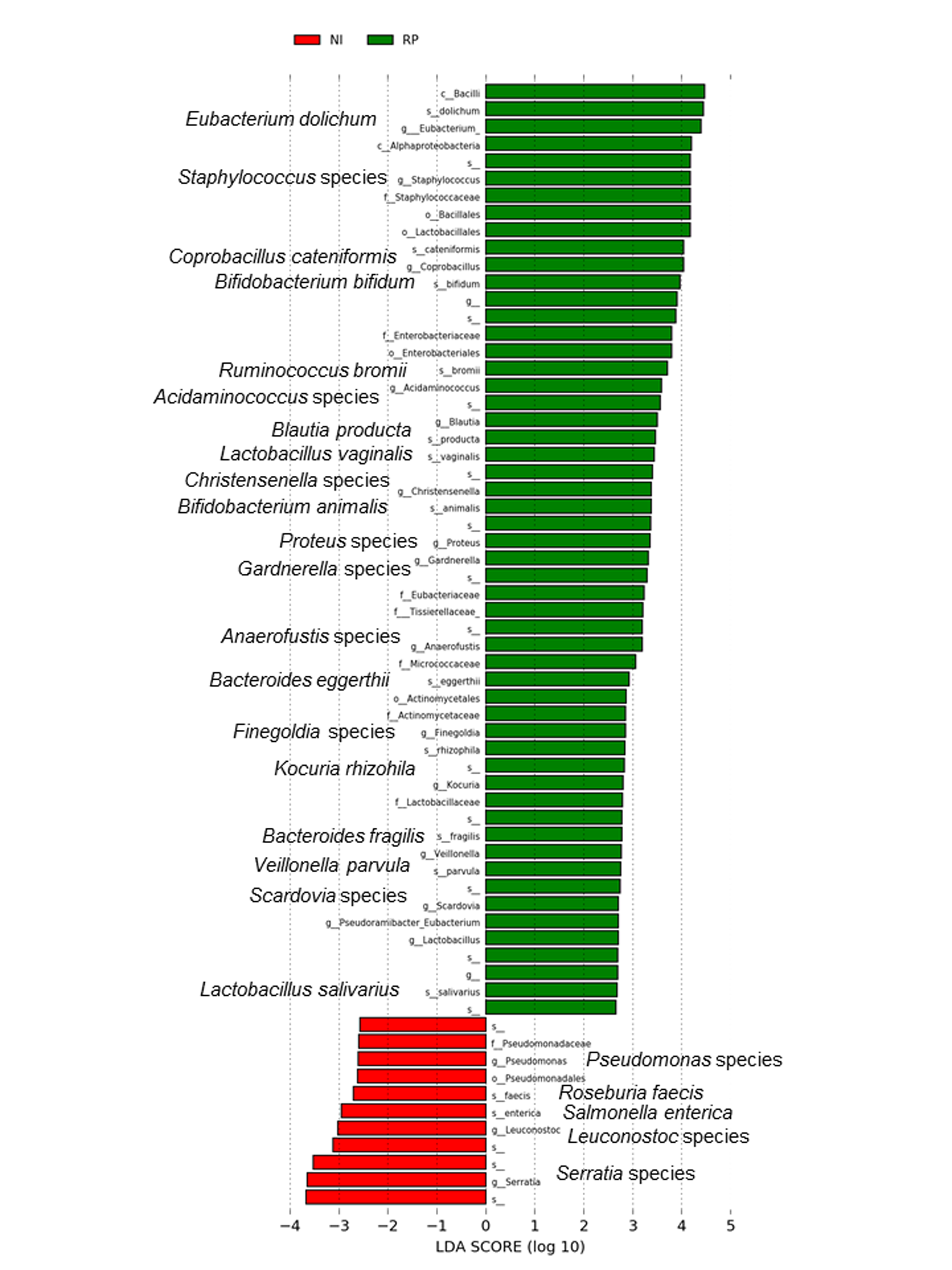

Supplement: S1 Fig — We analyzed metagenomic data of bacterial taxa using PICRUSt/LEfSe to assess major taxon differences between RP patients (RP) and normal individuals (NI). PICRUSt/LEfSe provided us with bar plots of prevalent biological features with the log LDA scores (effect sizes). In this chart, significantly enriched bacterial taxa in samples obtained from RP patients were exhibited by green bars. Significantly enriched bacterial taxa in samples obtained from normal individuals were exhibited by red bars. “p__”, “c__”, “o__”, “f__”, “g__”, and “s__” indicated phylum, class, order, family, genus, and species, respectively. Predominant species in RP patients and normal individuals were listed in Table 2. (TIF) [file pone.0203657.s001.tif]

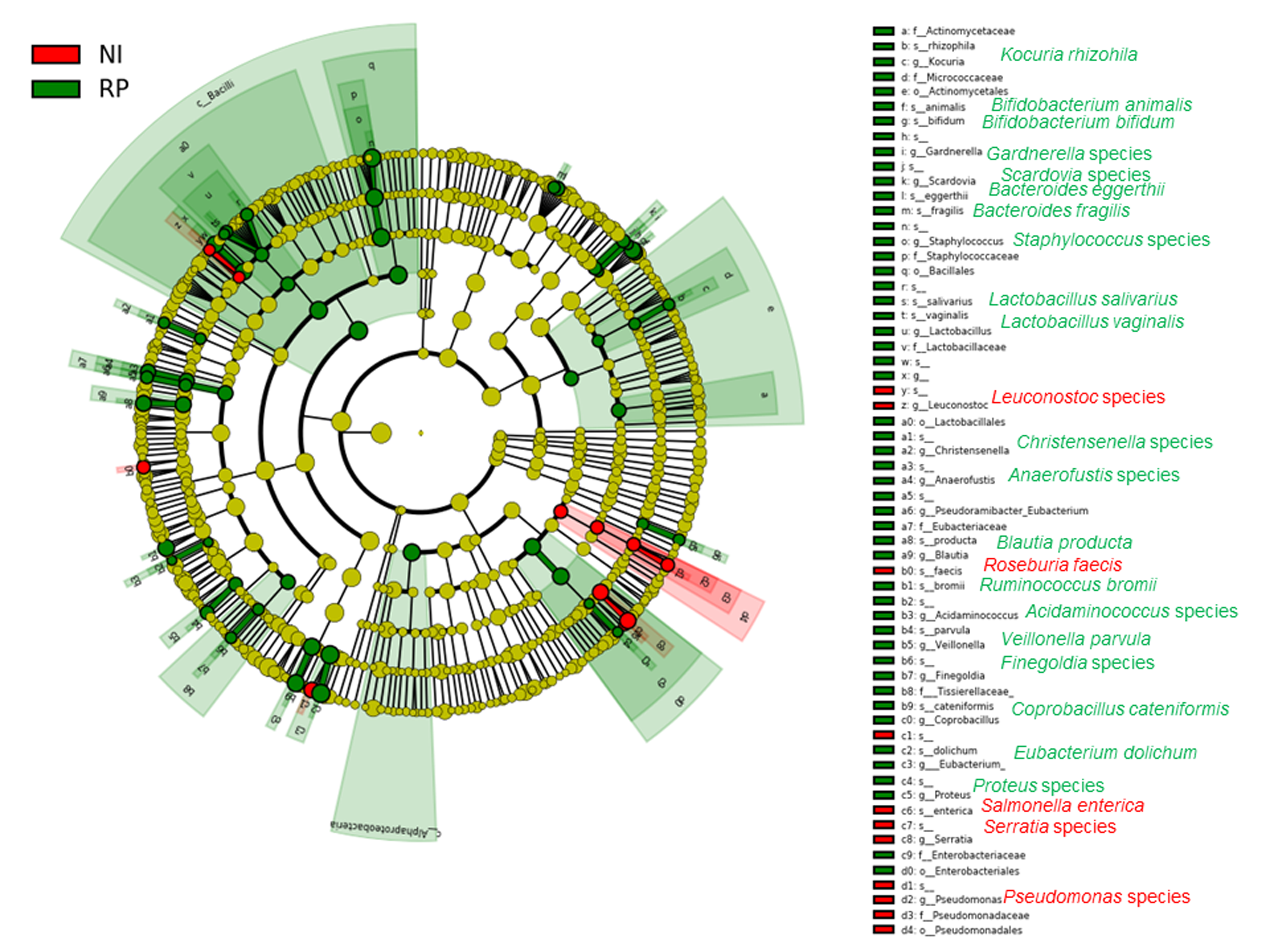

Supplement: S2 Fig — PICRUSt/LEfSe provided us with a cladogram of seven levels (from kingdom to species) from the same OTU table of S1 Fig. Circles ranged from the phylum (the innermost) to the species. Significantly enriched bacterial taxa in samples obtained from RP patients (RP) were exhibited by small green circles and green shadings. Significantly enriched bacterial taxa in samples obtained from normal individuals (NI) were exhibited by small red circles and red shadings. The circle sizes corresponded to the log LDA scores (effect sizes). This cladogram demonstrated that the class Bacilli, the order Actinomycetales, and several genera and species were abundant in RP patients. (TIF) [file pone.0203657.s002.tif]

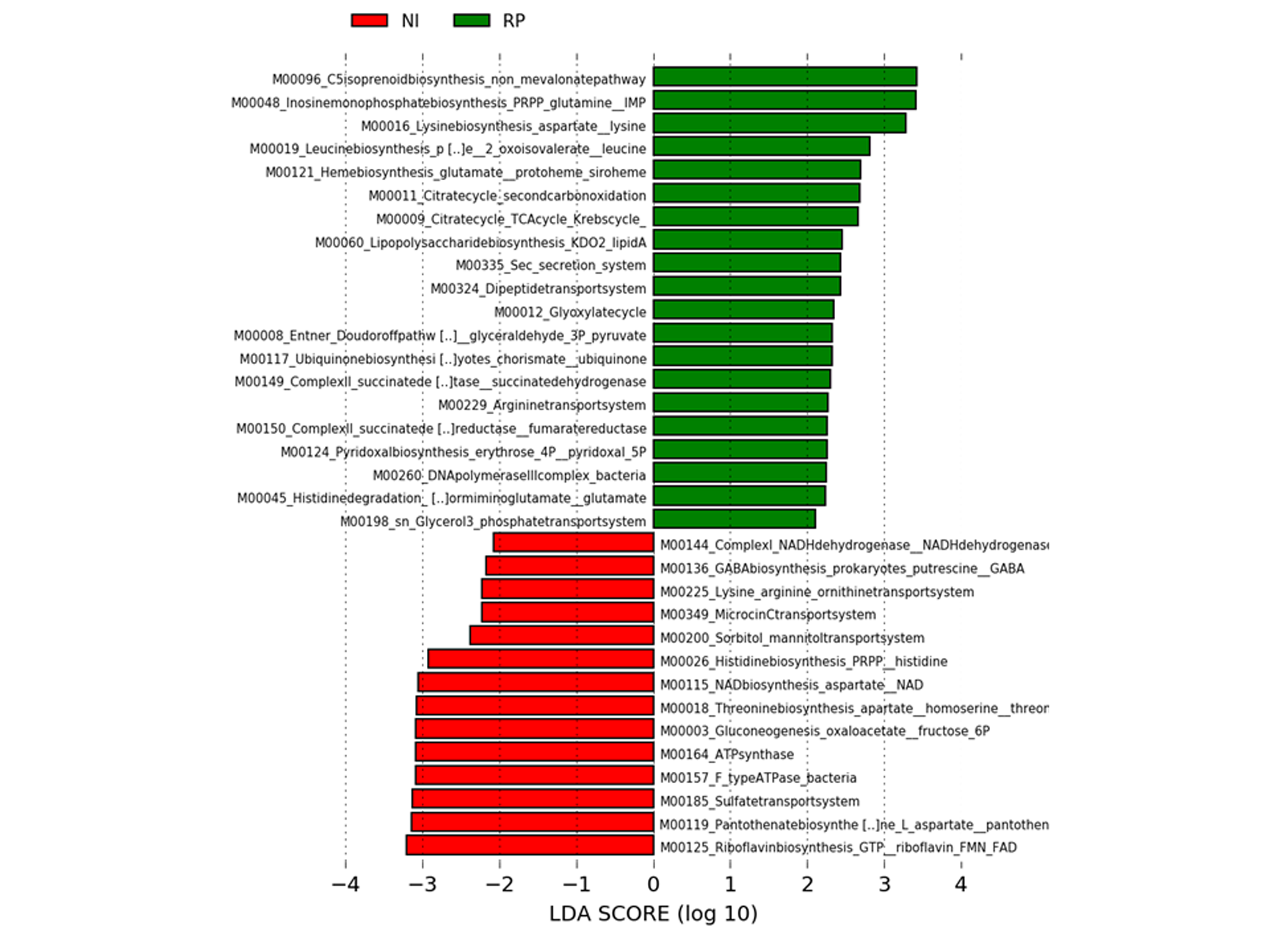

Supplement: S3 Fig — We assigned predicted gene functions according to the KEGG module database to identify predominant modules in RP patients (RP) and normal individuals (NI). Predominant KEGG modules in RP patients and normal individuals were listed in Tables 3 and 4, respectively. (TIF) [file pone.0203657.s003.tif]
